# Antitumor and Cytogenotoxic Activities of *Libidibia ferrea* Hydroalcoholic Extracts in Murine Breast Carcinoma

**DOI:** 10.1002/cbdv.202501032

**Published:** 2025-07-11

**Authors:** Diego Pereira de Menezes, Evaldo dos Santos Monção Filho, Atanara Alves de Sousa, João Pedro Alves Damasceno do Lago, Karla Darlianny Mourão Cavalcante, Fernanda Alves de Sousa Cavalcante, Irismara Sousa Silva, Marina Pinto Sanches, Semiramis Jamil Hadad do Monte, Ester Miranda Pereira, Paulo Michel Pinheiro Ferreira, Joao Marcelo de Castro e Sousa, Gerardo Magela Vieira Júnior, Dalton Dittz

**Affiliations:** ^1^ Postgraduate Program in Pharmaceutical Sciences, PPGCF Federal University of Piaui Teresina Brazil; ^2^ Postgraduate Program in Chemistry, PPGQ Federal University of Piaui Teresina Brazil; ^3^ Postgraduate Program in Health Sciences, PPGCS Federal University of Piaui Teresina Brazil; ^4^ Biomedicine Course, Higher Education Association of Piaui AESPI Teresina Brazil; ^5^ Postgraduate Program in Pharmacology Federal University of Piaui Teresina Brazil; ^6^ Biological Science School Federal University of Piaui Teresina Brazil; ^7^ Department of Physiology and Pharmacology Federal University of Pernambuco Recife Brazil; ^8^ Postgraduate Program in Technologies Applied to Animals of Regional Interest, PPGTAIR Federal University of Piaui Teresina Brazil; ^9^ Immunogenetics and Molecular Biology Laboratory LIB Federal University of Piaui Teresina Brazil; ^10^ Department of Biophysics and Physiology Federal University of Piaui Teresina Brazil; ^11^ Department of Biochemistry and Pharmacology Federal University of Piaui Teresina Brazil

**Keywords:** antioxidant properties, antitumor activity, breast cancer, *Libidibia ferrea*, phenolic compounds

## Abstract

Breast cancer is the most prevalent cancer type among women worldwide, and there is a critical need for more effective and safer treatments, especially for aggressive and therapy‐resistant subtypes. *Libidibia ferrea*, a native plant from Brazil, exhibits anti‐inflammatory, antimicrobial, antioxidant, and antitumor properties. This study evaluated the antioxidant activity and antitumor effects of hydroalcoholic extracts from the leaves (HAFL) and fruits (HAFR) of *L. ferrea* in murine breast carcinoma models. Chemical analysis revealed several phenolic compounds, including gallic and ellagic acids. HAFR showed three times more total phenolic content than HAFL and exhibited higher antioxidant, cytotoxic, and genotoxic activities, inducing DNA damage and cell death in MDA‐MB‐231 cells. In a 4T1 murine model, HAFR reduced tumor growth by 94% at doses of 0.3 and 3.0 g/kg without affecting body or liver weight. These findings suggest HAFR's potential as a therapeutic candidate for breast cancer treatment.

## Graphical abstract

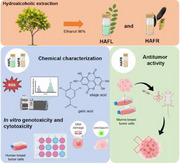

## 1 Introduction

A major challenge in addressing breast carcinoma lies in its genetic heterogeneity, which defines molecular subtypes of the disease that influence the prognosis and therapeutic responses of patients [1, 2]. Given these challenges, natural products have gained prominence as a source of bioactive compounds with therapeutic potential, including antitumor activity.

**FIGURE 1:**
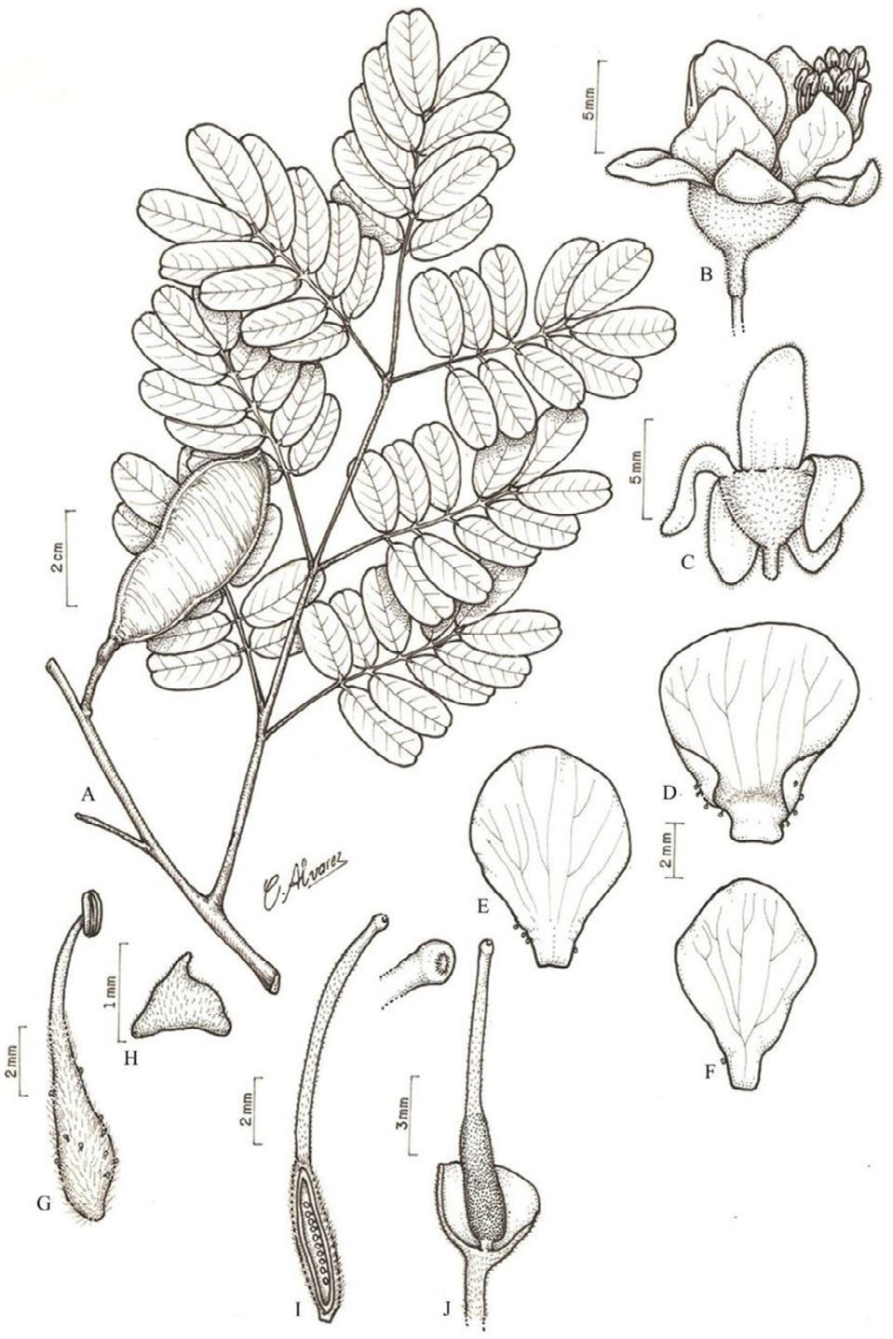
*Libidibia ferrea* (Mart. ex Tul.) L.P. Queiroz. (A) Branch with fruit. (B) Flower. (C) Calyx. (D) Standard. (E) Wing. (F) Keel. (G) Stamen. (H) Bracteole. (I) Ovary in longitudinal section. (J) Hypanthium in a longitudinal section showing the insertion of the gynoecium. Reproduced from Rodrigues et al. [9], licensed under CC BY 4.0.

Among promising plants, *Libidibia ferrea* (Figure 1), native to the Brazilian Northeast, has been traditionally used to treat cardiovascular diseases, diabetes, rheumatism, and as an anti‐inflammatory and analgesic [3]. Research has revealed its antiproliferative properties, although limited to specific malignancies. Azevedo et al. [4] demonstrated that ethanolic extracts of *L. ferrea* pods inhibit gastric adenocarcinoma cell migration. Additionally, the fruit extract exhibits apoptotic activity in colorectal cancer cells, marked by caspase‐3 activation, reduced Bcl‐2 levels, AKT pathway inhibition, and downregulation of the EGF receptor [5]. These effects have been attributed to phenolic compounds such as gallic acid, a cytotoxic and antitumor agent, and ellagic acid, known for its antioxidant and antiproliferative properties [6, 7]. Beyond its antitumor potential, ethanolic bark extracts of *L. ferrea* have demonstrated notable antioxidant and anti‐inflammatory activities in acute inflammation models, enhancing enzymatic and non‐enzymatic antioxidant defenses while reducing oxidative metabolites [8]. Despite these findings, there is a critical need to investigate the cytotoxicity and underlying mechanisms of *L. ferrea* extracts in other cancer types, such as breast cancer. Exploring these mechanisms, including the role of phenolic compounds and oxidative stress modulation, is essential to identify bioactive components and clarify their contributions to antitumor activity. Based on these reports, the hydroalcoholic extracts from fruit and leaves of *L. ferrea* were chemically characterized and evaluated for their antioxidant, genotoxicity, cytotoxicity, and antitumor effects in a murine breast carcinoma model. This study aims to improve the knowledge of the mechanisms of action and therapeutic potential of *L. ferrea*.

## 2 Results and Discussion

### 2.1 Chemical Characterization of L. Ferrea by High‐Performance Liquid Chromatography With Diode Array Detector and Direct Insertion With an Electrospray Ionization Source and an Ion Trap Analyzer Mass Spectrometry

The hydroalcoholic extracts from the fruits (HAFR) and leaves (HAFL) were analyzed by high‐performance liquid chromatography with diode array detector (HPLC‐DAD) and confirmed the presence of gallic and ellagic acid, compared with standards, in comparison with their respective retention time and ultraviolet (UV) spectrum as shown in Figure S1.

**FIGURE 2:**
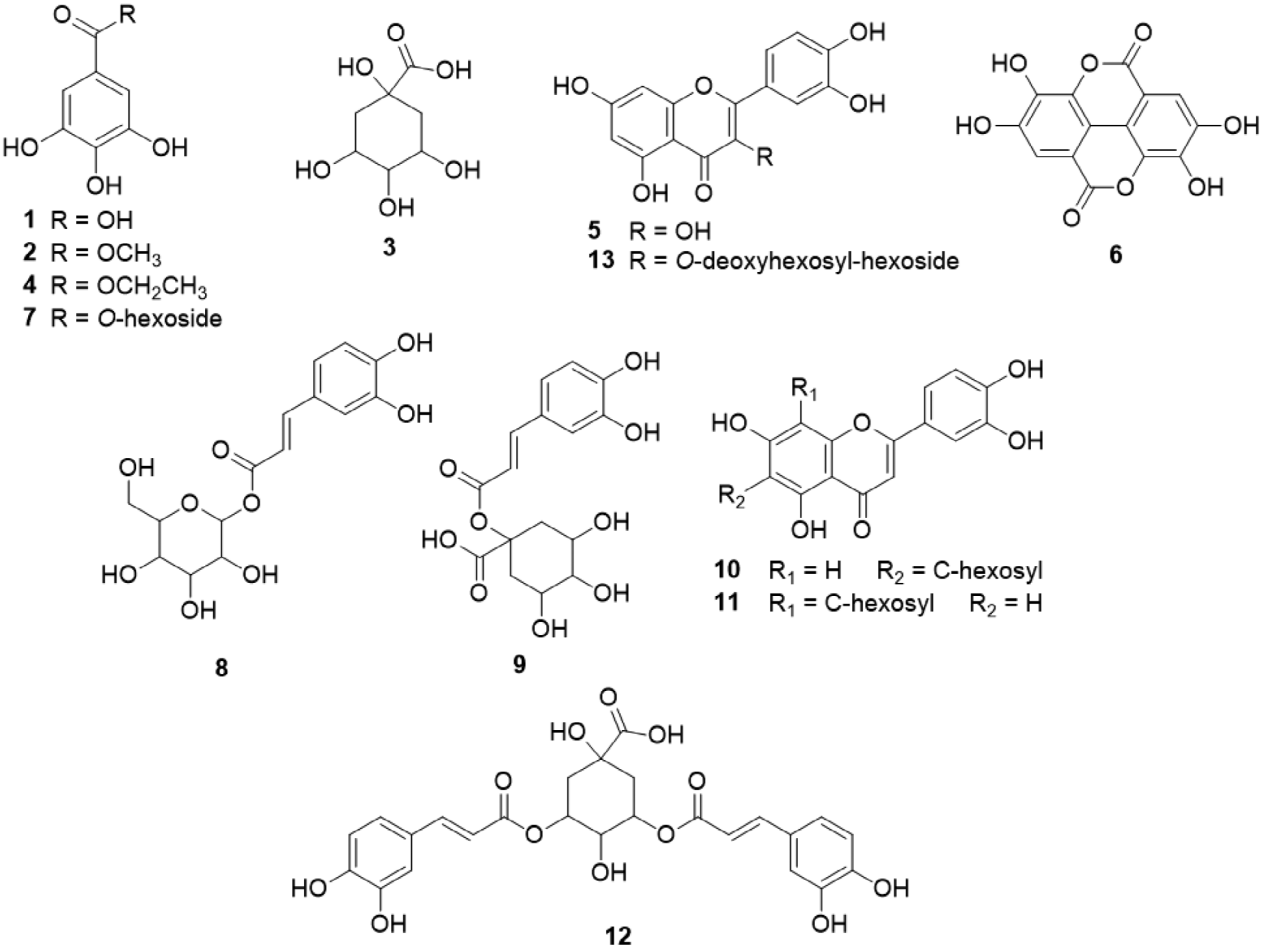
Structure of annotated compounds by direct insertion with an electrospray ionization and tandem mass spectrometry (DI‐ESI‐MS/MS) in hydroalcoholic extracts of *L. ferrea* in negative mode.

Both extracts were also analysed by direct insertion with an electrospray ionization source and an ion trap analyzer mass spectrometry (DI‐ESI‐IT‐MS^n^), and 13 compounds were annotated, including gallic and ellagic acids, flavonoids, hydroxybenzoic and quinic acid derivatives. Their structural propositions are shown in Figure 2, and their respective deprotonated molecular mass, fragments, and the presence in each observed extract are described in Table 1.

**TABLE 1:** Molecular mass, fragments, and annotation of fruits (HAFR) and leaves (HAFL) extracts from L. ferrea compounds by direct insertion with an electrospray ionization source and an ion trap analyzer tandem mass spectrometry (DI‐ESI‐MS/MS^n^) in negative mode.

Compound	[M‐H]^−^	MS/MS^n^ (relative abundance) Base peak in bold	HAFL	HAFR	Annotation	References
**1**	169	124.8 (**100**)	+	+	Gallic acid	ESCOBAR‐AVELLO et al. 2019
**2**	183	167.8 (**100**), 123.9 (28)	−	+	Methyl gallate	SOBEH et al. 2019
**3**	191	172.8 (18), 170.7 (48), 126.9 (**100**), 110.9 (61)	−	+	Quinic Acid	BASTOS et al. 2007
**4**	197	168.7 (**100**), 124.8 (15) (MS^3^: 169): 124.7 (**100**)	−	+	Ethyl gallate	WYREPKOWSKI et al. 2014
**5**	301	282.9 (57), 253.1 (55), 178.9 (**100**), 150.8 (56) (MS^3^: 179): 150.7 (**100**) (MS^4^: 151): 106.9 (**100**)	+	−	Quercetin	SOBEH et al. 2019
**6**	301	283.9 (17), 256.8 (**100**), 228.8 (48), 184.8 (65)	−	+	Ellagic acid	WYREPKOWSKI et al. 2014
**7**	331	270.8 (47), 240.8 (36), 210.8 (21), 168.8 (**100**) (MS^3^: 169): 124.8 (**100**)	−	+	Galoyl‐hexoside	ESCOBAR‐AVELLO et al. 2019
**8**	341	178.9 (**100**), 160.9 (39), 142.9 (25), 118.9 (19)	−	+	Caffeoylhexoside	BASTOS et al. 2007
**9**	353	178.8 (37), 172.8 (**100**) (MS^3^: 173): 154.8 (67), 110.8 (**100**)	−	+	Caffeoylquinic acid	BASTOS et al. 2007
**10/11**	447	428.9 (16), 356.9 (84), 326.9 (**100**), 284.9 (13) (MS^3^: 327): 298.9 (**100**), 283.9 (11) (MS^4^: 299): 270.8 (40), 254.8 (**100**), 241.8 (30), 198.8 (32)	+	−	Orientin/Isoorientin	ZHANG et al. 2018
**12**	515	340.9 (**100**), 178.9 (7) (MS^3^: 341): 178.8 (**100**), 160.7 (19), 142.8 (27) (MS^4^: 179): 130.8 (92), 118.7 (72), 112.8 (**100**)	−	+	Dicaffeoylquinic acid	BASTOS et al. 2007
**13**	609	342.9 (6), 300.8 (**100**), 270.8 (9) (MS^3^: 301): 270.8 (88), 254.8 (60), 178.8 (87), 150.8 (**100**)	+	−	Quercetin‐*O*‐deoxyhexosyl‐hexoside	BASTOS et al. 2007

Spectrometric analyses showed that both extracts contain gallic acid, while ellagic acid was exclusively detected in HAFR. However, the HPLC‐DAD analysis showed these compounds in HAFR and HAFL in comparison with standards. In DI‐MS, an ion could be in competition, which can occur at the ionization source or also in the detection system [10]. The presence of quercetin (*m/z* 301) in HAFL may have suppressed the ellagic acid (*m/z* 301) detection due to their similar deprotonated molecular mass. Additional compounds, such as methyl/ethyl gallate and caffeoylquinic acid derivatives, were predominantly present in HAFR. The higher phenolic content in HAFR supports its antioxidant and antitumoral potential. Phenolic compounds are known for their redox activity, neutralizing free radicals, and reducing oxidative stress, as shown in previous studies on *L. ferrea* bark extracts [8]. Such properties are crucial, as oxidative stress plays a significant role in cancer development. Gallic acid is recognized for its ability to induce apoptosis, regulate pro‐ and anti‐apoptotic proteins, and inhibit metastasis in tumor models [7]. Ellagic acid, a derivative of gallic acid, exhibits antiproliferative and antioxidant effects by activating caspase‐3 and reducing reactive oxygen species [6]. These findings highlight the phytochemical diversity of *L. ferrea*, including the presence of bioactive compounds such as gallic and ellagic acids, which aligns with its reported bioactivity in other studies.

### 2.2 Total Phenolic Content and Antioxidant Activity

The total phenolic content (TPC) for the HAFR contained 487.62 ± 10.3 mg/g, almost three times higher than the 162.54 ± 8.4 mg/g in the HAFL, expressed in gallic acid equivalent milligrams per extract grams (GAE/g). The HAFR demonstrated a great amount of phenolic content, compared to the literature, and both were evaluated for their antioxidant activity. This amount of phenolic compounds in the fruit extract of *L*. *ferrea* may contribute significantly to the antioxidant properties [11]. The evaluation of the antioxidant capability of HAFR and HAFL extracts was developed by the colorimetric antiradical capacity, 1,1‐diphenyl‐2‐picrylhydrazyl (DPPH) assay method [12].

The HAFR and HAFL showed values of efficient concentration (EC_50_) of 223.2 and 428.9 µg/mL, respectively, with a better value for HAFR. Notably, when compared to the reference antioxidant compound, butylated hydroxytoluene (BHT) with EC_50_ 69.34 µg/mL, is not superior, but HAFR can be effective in higher concentrations. This antioxidant property aligns with the high concentration of total phenols in HAFR compared to HAFL, as phenolic compounds are known for their redox capacity, contributing to peroxide decomposition, free radical neutralization, and adsorption [13].

### 2.3 Cytotoxicity and Genotoxicity

**FIGURE 3:**
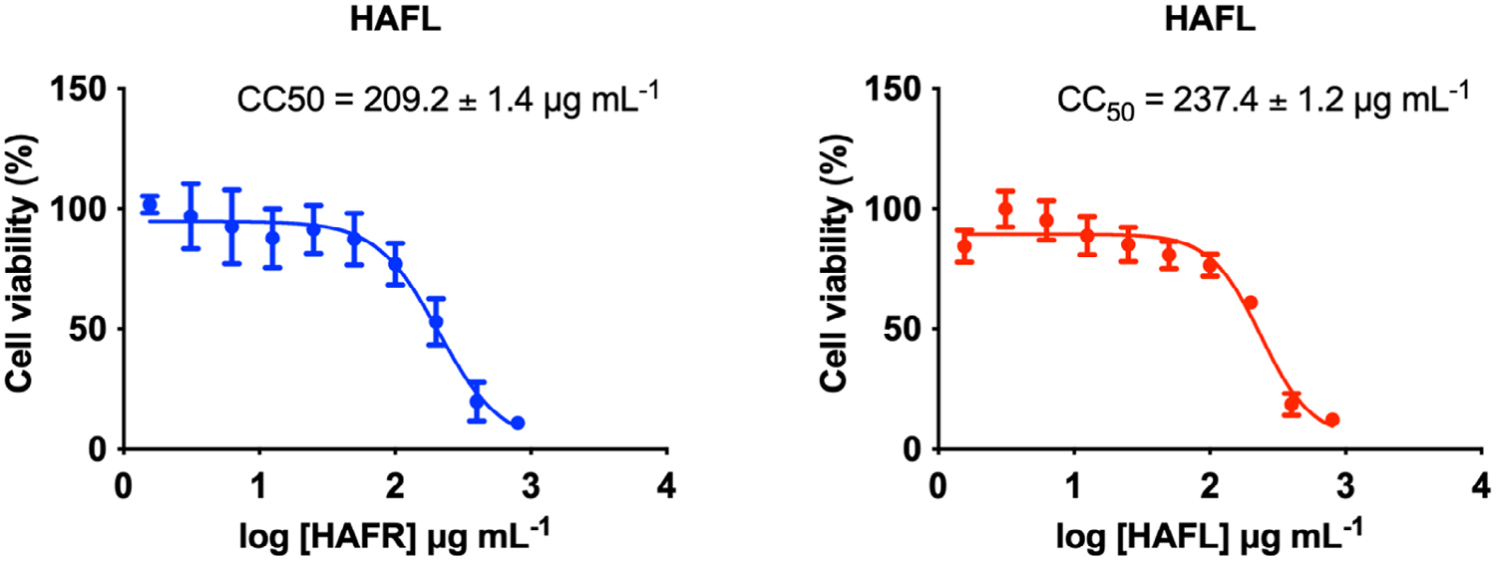
Cytotoxic concentration of hydroalcoholic extracts from the fruits (HAFR) and leaves (HAFL) in MDA‐MB‐231 cells. The triple‐negative breast cancer cell line MDA‐MB‐231 was exposed to HAFR and HAFL at concentrations up to 800 µg/mL for 72 h. Cell viability was measured using the MTT assay at 570 nm. Results are expressed as the mean ± standard error of the mean, and 50% cytotoxic concentration (CC_50_) values were determined by nonlinear regression from three independent experiments.

The cytotoxic and genotoxic effects of HAFR and HAFL extracts were assessed against the triple‐negative breast cancer cell line, MDA‐MB‐231. Both extracts significantly reduced cell viability at concentrations above 100 µg/mL, with 50% cytotoxic concentration (CC_50_) values of 209.2 ± 1.4 µg/mL for HAFR and 237.4 ± 1.2 µg/mL for HAFL (Figure 3).

**FIGURE 4:**
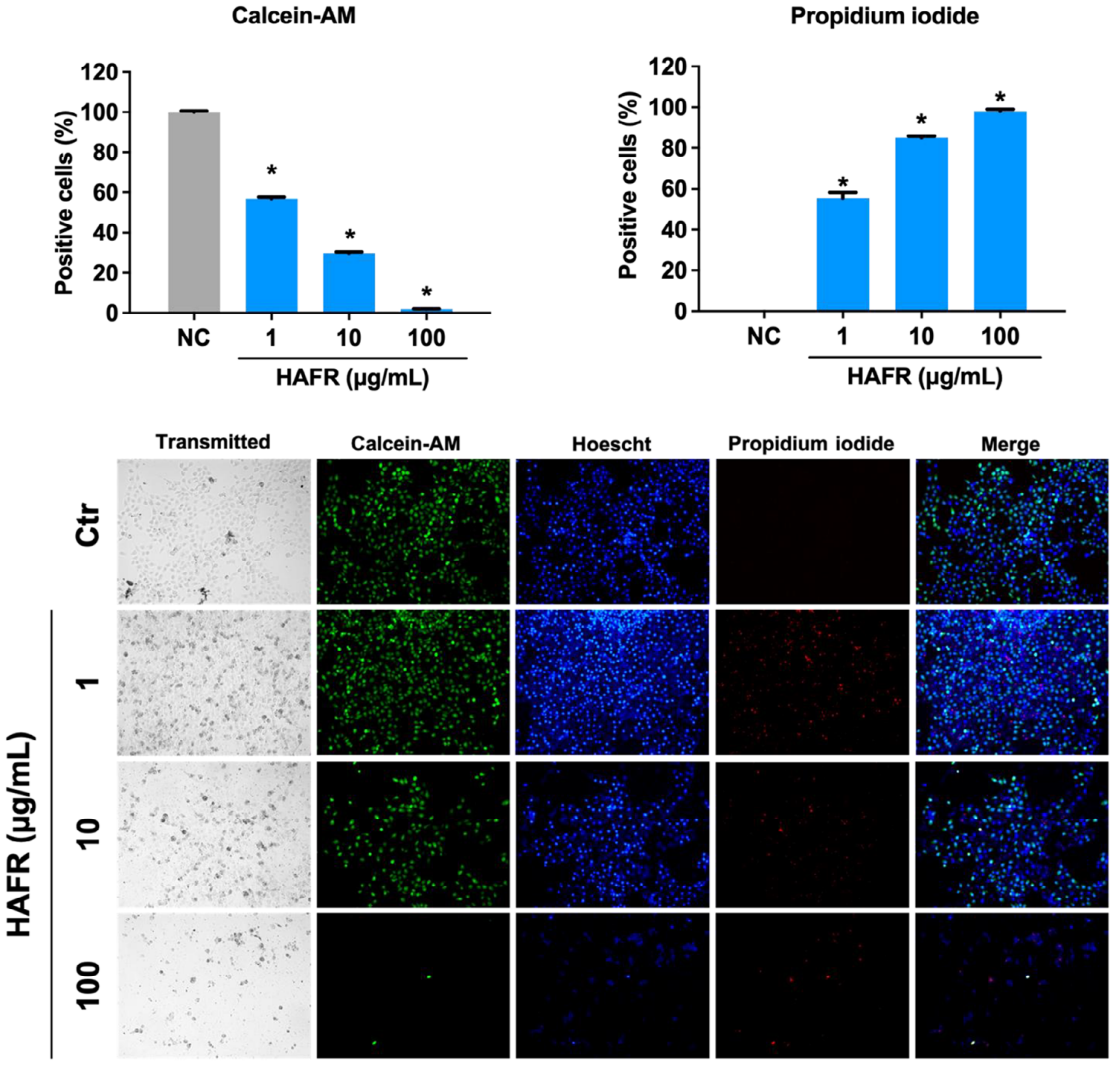
Effect of hydroalcoholic extracts from the fruits (HAFR) on MDA‐MB‐231 cells' viability. Cells were exposed to HAFR (1, 10, and 100 µg/mL) for 72 h, and the fluorescence of Calcein‐AM, Hoechst, and propidium iodide was assessed by fluorescence microscopy. (A) The percentages of cell viability and cell death were quantified based on the fluorescence intensity of Calcein‐AM and propidium iodide, respectively, relative to the negative control group (NC). Results are expressed as mean ± standard error. *p < 0.05, one‐way analysis of variance (ANOVA), Bonferroni post‐test, compared to NC. (B) Representative panel of cell fluorescence. The scale bar represents 100 µm.

The assessment of MDA‐MB‐231 cell death revealed that HAFR, even at the lowest concentration of 1 µg/mL, significantly reduced cell viability compared to the negative control group (*p < 0.05). There was a marked reduction in viable cells, with Calcein‐AM staining indicating 57% at 1 µg/mL, 30% at 10 µg/mL, and only 2% at 100 µg/mL. Additionally, a significant increase in propidium iodide (PI) staining, corrected by the total number of cells in each group, was observed compared to the negative control group (*p < 0.05) for HAFR 1 µg/mL (55%), 10 µg/mL (85%) and 100 µg/mL (98%) (Figure 4).

**FIGURE 5:**
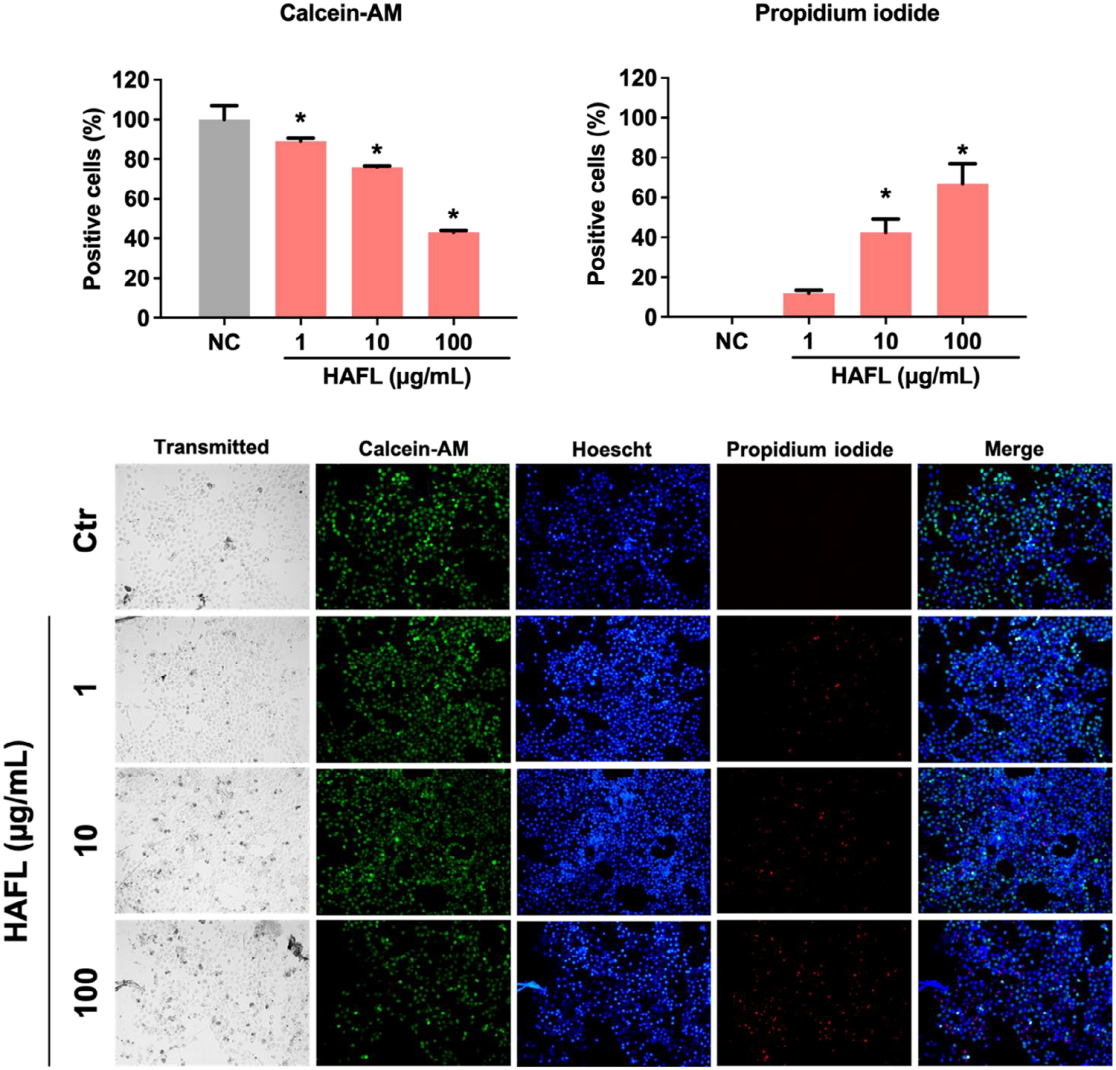
Effect of hydroalcoholic extracts from the leaves (HAFL) on MDA‐MB‐231 cells' viability. Cells were exposed to HAFL (1, 10, and 100 µg/mL) for 72 h, and the fluorescence of Calcein‐AM, Hoechst, and Propidium Iodide was assessed by fluorescence microscopy. (A) The percentages of cell viability and cell death were quantified based on the fluorescence intensity of Calcein‐AM and propidium iodide, respectively, relative to the negative control group (NC). Results are expressed as mean ± standard error. *p < 0.05, one‐way analysis of variance (ANOVA), Bonferroni post‐test, compared to NC. (B) Representative panel of cell fluorescence. The scale bar represents 100 µm.

Treatment with HAFL also resulted in a significant increase in PI staining at 10 µg/mL (42%) and 100 µg/mL (69%) compared to the control group (*p < 0.05). At a concentration of 100 µg/mL, there was a reduction of 43% in cell viability compared to the control (*p < 0.05), as shown by Calcein‐AM staining (Figure 5).

Although the cytotoxic concentration of both extracts is similar, HAFR reduced Calcein‐AM staining and the total number of cells at lower concentrations than HAFL. The lower intensity of PI fluorescence with increasing HAFR concentrations likely reflects a reduction in cell numbers, highlighting its stronger cytotoxic potency. This discrepancy may stem from the differences in the assays used: the MTT assay indirectly measures cell viability based on mitochondrial enzyme activity, while Calcein‐AM and PI staining directly assess cell viability through cytoplasmic esterase activity and membrane integrity. Therefore, while mitochondrial activity is similar between the treatments, HAFR causes more significant membrane damage and loss of esterase activity, leading to greater cell death.

**FIGURE 6:**
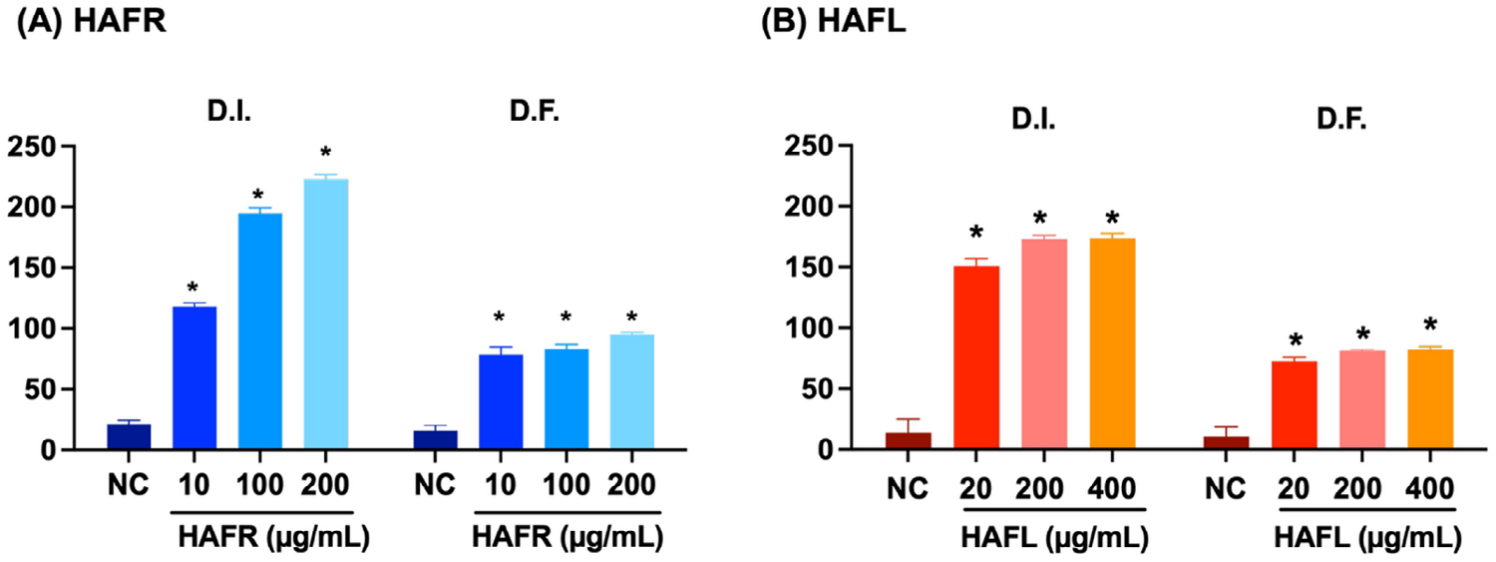
Damage indices and damage frequency caused by hydroalcoholic extracts from the leaves (HAFL) and fruits (HAFR) in MDA‐MB‐231 cells. Damage indices (D.I.) and Damage Frequency (F.D.) for HAFR (A) and HAFL (B) are expressed as mean ± standard error. *p < 0.05, two‐way analysis of variance (ANOVA), Tukey's post‐test compared to negative control (NC).

Both extracts caused genotoxic damage to MDA‐MB‐231 cells, with DNA damage observed in a concentration‐dependent manner, as reflected by the damage index (DI) and damage frequency (DF). At higher concentrations, HAFR induced greater DI and DF compared to HAFL. Specifically, at 20 µg mL^−1^, HAFL increased DI and DF by 11‐fold and 10‐fold, respectively, compared to the control (*p < 0.05). At 400 µg mL^−1^, HAFL caused even more pronounced genotoxic effects (Figure 6).

At the highest concentrations of HAFL and HAFR, there was a greater predominance of level 2 and 3 damage. In contrast, only HAFR caused level 4 damage to the nucleolus of MDA‐MB‐231 cells (Table 2 and Figure S2).

**TABLE 2:** Percentage of genotoxic damage of hydroalcoholic extracts from the fruits (HAFR) and leaves (HAFL) in MDA‐MB‐231

Damage level	Damage percentage					
	HAFR (µg/mL)			HAFL (µg/mL)		
	10	100	200	20	200	400
0 – 1^[a]^	32	22	6	36	24	21
2 – 3^[b]^	65	71	75	64	76	79
4^[c]^	3	7	19	0	0	0

^[a]^ No damage to low damage: a small tail is visible, with minimal DNA migration.

^[b]^ Moderate to high damage: the tail contains a significant portion of the DNA, leaving only a small amount in the nucleus.

^[c]^ Severe damage: nearly all the DNA has migrated into the tail, with the nucleus barely visible.

These findings align with previous studies demonstrating the cytotoxic activity of *L. ferrea* fruit extract in colorectal carcinoma cells (HT‐29) [5] and gastric [4]. In contrast, hydroalcoholic extracts of *L. ferrea* fruit did not induce genotoxic damage in gastric adenocarcinoma cells, attributing this to the extract's antioxidant properties [4]. These findings suggest that factors such as extraction methods, composition, and cell type are critical in determining whether the effects are protective or cytotoxic.

### 2.4 Antitumor Activity in the Triple‐Negative Breast Cancer Model

**FIGURE 7:**
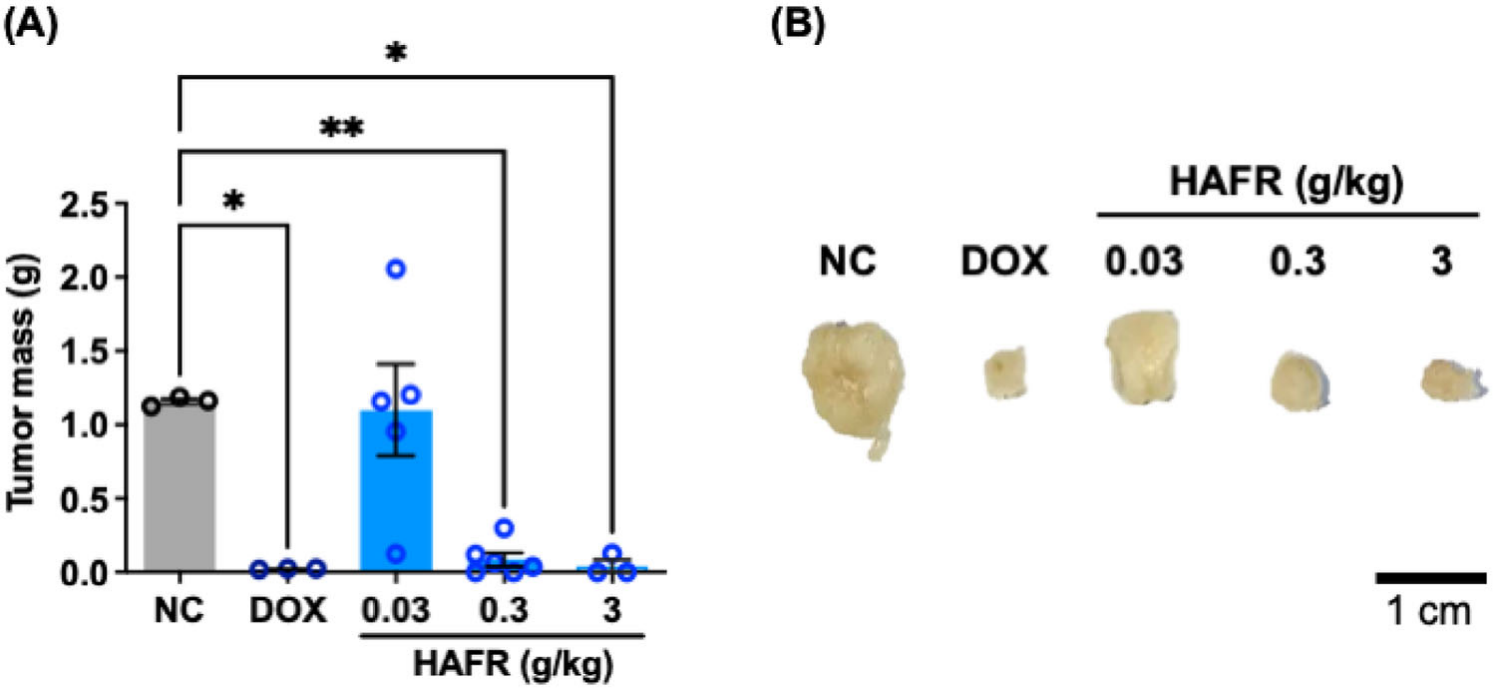
Antitumoral activity of hydroalcoholic extracts from the fruits (HAFR) in the triple‐negative breast cancer (TNBC) model. 4T1 cells (5 × 10⁶ cells/100 µL) were inoculated into the mammary gland of the animals. The animals were treated intraperitoneally with doxorubicin (DOX) at 37.5 mg/kg in four doses, administered every three days, or with HAFR for 21 days. (A) Tumor mass is expressed as mean ± standard deviation (*p < 0.05; **p < 0.01, analysis of variance [ANOVA] followed by Tukey's post hoc test). (B) Representative tumors from the experimental groups (*n* = 7 animals/group).

Considering the chemical characterization and in vitro results, HAFR was selected to proceed with the assessment of its antitumor effect in the triple‐negative breast cancer (TNBC) model (Figure 7). A tumor weight reduction of approximately 94% was observed in animals treated with doses of 0.3 and 3.0 g/kg of HAFR compared to the negative control group (*p < 0.05). The positive control, doxorubicin at 37.5 mg/kg, showed a 75% reduction in tumor mass compared to the negative control (*p < 0.05). Considering that the antitumor activity of HAFR 0.3 or 3 g/kg was similar to doxorubicin, suggesting that HAFR's antitumor efficacy is comparable to an established breast cancer treatment.

In a colorectal carcinoma model, the crude fruit extract of *L. ferrea* combined with oxaliplatin, delivered in a formulation with extracellular vesicles derived from M1 macrophages, modulated the tumor microenvironment and enhanced M2 macrophage activity [14]. Conversely, purified fractions from the crude fruit extract of *L. ferrea* did not significantly reduce tumors in sarcoma 180‐bearing animals [15]. Together with our results, we can infer that the antitumor activity of *L. ferrea* fruit extracts is related to their extraction method and the type of tumor.

### 2.5 Body and Spleen Mass and Hepatic Malondialdehyde Levels

**FIGURE 8:**
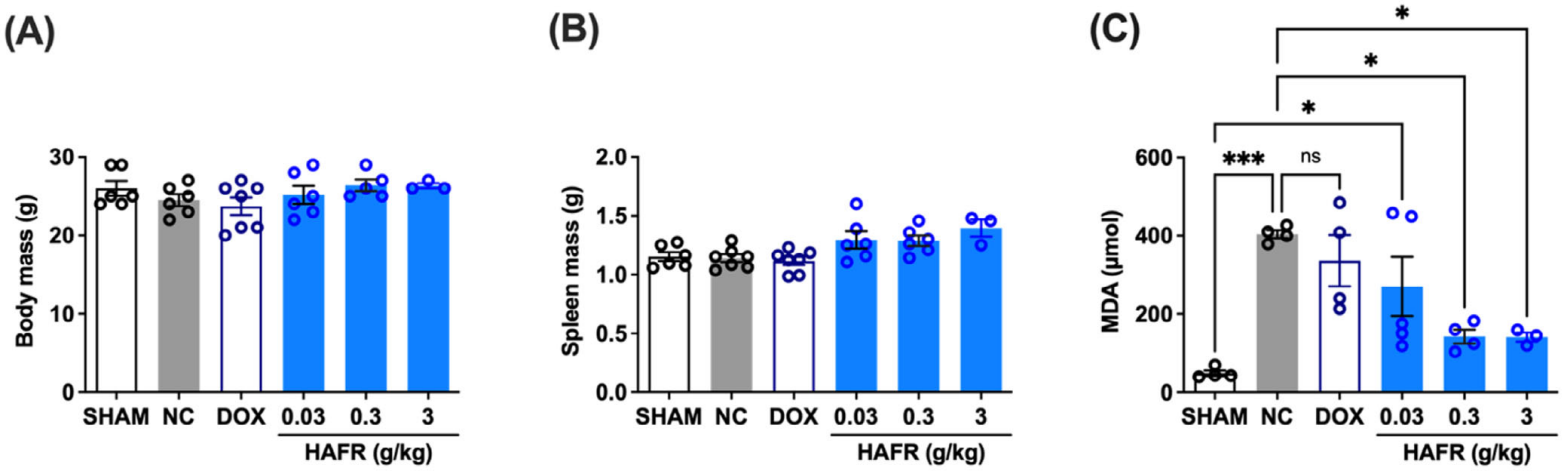
Body and spleen mass and hepatic malondialdehyde levels of hydroalcoholic extracts from the fruits (HAFR)‐treated triple‐negative breast cancer (TNBC) mice of HAFR‐treated TNBC mice. Body (A) and spleen (B) masses and malondialdehyde (C) concentrations were expressed as mean ± standard error (*p < 0.05, ***p < 0.001, analysis of variance [ANOVA] followed by Tukey's post hoc test).

After 21 days of treatment of animals with 4T1 breast tumors with HAFR, no significant changes were observed in the total weight and liver weight of the animals (Figures 8A and 8B). Tumor induction promoted an 83% increase in hepatic malondialdehyde (MDA) levels (*p < 0.05, compared to the Sham). In turn, treatment with HAFR at doses of 0.3 and 3 g/kg reduced hepatic MDA levels by approximately 68% (*p < 0.05) when compared to the negative control group. There was no significant reduction in MDA levels in animals treated with doxorubicin 37.5 mg/kg or HAFR 0.03 g/kg (Figure 8C). The reduction of MDA levels in the blood of mice with acute inflammation was also observed when they were treated with hydroalcoholic fractions (20%–80% ethanol) of *L. ferrea* fruit at doses of 50 and 200 mg/kg [3]. The reduction of MDA in an in vivo system further supports the antioxidant property described here for DPPH radical scavenging and highlights the hepatoprotective role of HAFR. The absence of changes in total body weight and liver weight, along with the reduction in hepatic lipid peroxidation, supports the safety profile for the systemic use of HAFR. However, further studies, such as the determination of lethal doses, biochemical parameters, and histological evaluation, are necessary to confirm this safety.

## 3 Conclusions

The HAFL and HAFR of *L. ferrea* contain phenolic compounds, including gallic and ellagic acids, and this composition can be associated with the antioxidant activity of the extracts. In a triple‐negative breast cancer cell line, both extracts exhibited cytotoxic effects and induced DNA fragmentation, with these effects being more pronounced for HAFR. These activities contribute to the antitumor effect of HAFR in a murine breast carcinoma model, where the treatment showed no toxicity for the evaluated parameters. The limitations are reflected in the need for a molecular investigation of the interaction of the investigated compounds and the activation pathways of cell death, especially apoptosis, and identification of the major compound of the extracts.

## 4 Experimental

### 4.1 Obtention of Hydroalcoholic Extracts From *L. ferrea*

The HAFR and HAFL of *L. ferrea* were supplied by Phytobios Pesquisa, Desenvolvimento e Inovação Ltda (Parnaíba, Piauí, Brazil), under a service agreement. The extraction process was conducted by Phytobios using standardized and validated procedures. The authors opted to outsource the extraction due to the company's infrastructure and experience in obtaining phytochemical‐rich extracts. Extracts were obtained by exhaustive maceration at room temperature for 24 h with ethanol 96% (v/v), facilitating the extraction of polar compounds. After ethanol evaporation, extracts were concentrated in a rotary evaporator and on a heating plate at 45°C. The yield of the HAFL extract was 5.8%–6.2%, and that of the HAFR was 3.2%–3.5%. The methodology was selected for its efficiency in extracting a broad phytochemical profile [16]. Specimens of *L. ferrea* were collected in August/2023 at coordinates 03°21′49.70″S, 41°46′28.80″W (−3.3638, −41.7747), in a native Caatinga area in northern Piauí, Brazil. The collection and identification were carried out by a professional specialized in the regional flora from Phytobios. A voucher specimen (HDELTA 1819) was deposited at the Herbarium Delta (HDELTA). Access to genetic resources was registered in the National System for the Management of Genetic Heritage and Associated Traditional Knowledge (SisGen), under registration code A8B0495.

### 4.2 Determination of TPC

The TPC of the extracts was determined using the Folin‐Ciocalteu method performed in triplicate [17]. Both extracts (5 mg) were dissolved separately in 10 mL of methanol (Labsynth—São Paulo, Brazil), and the aqueous 15% sodium carbonate solution was prepared and stored. An aliquot of 100 µL of the extract solution was added to a volumetric flask (10 mL), with 500 µL of Folin‐Ciocalteu reagent (Dinâmica—São Paulo, Brazil), and 5 mL of distilled water. After 1 min of stirring, 2 mL of 15% sodium carbonate solution (Labsynth—São Paulo, Brazil) was also added, and the solution was stirred again for 30 s. The volume of 10 mL was made up with distilled water and allowed to react for 2 h. The blank was performed using the same described methodology, replacing the extract solution with 100 µL of methanol. The absorbance was measured in a Lambda 25 UV/Vis spectrometer (PerkinElmer—São Paulo, Brazil) at a wavelength of 750 nm.

The TPC was determined through interpolation of the absorbance against the analytical curve constructed using concentration in the range of 10–350 µg/mL of the gallic acid standard (Sigma Aldrich—Poole, UK), and it was expressed as *A = 0.1185c − 0.0453A* (*r* = 0.999), where “c” is the concentration of gallic acid and “A” is the absorbance. The results were expressed as milligrams of gallic acid equivalent per gram of extract (mg GAE g^−1^).

### 4.3 HPLC Analysis

The HAFR and HAFL extracts (21 mg each) were pre‐treated using an AccuBond II‐C18 solid‐phase extraction (500 mg^3^/mL) cartridge (Agilent Technologies—CA, US), eluted with 5 mL of methanol per sample. The dried extracts (5 mg of each sample) and gallic acid and ellagic acid (Sigma Aldrich—Poole, UK) standards (1 mg) were dissolved in 2 mL of a MeOH/H_2_O mixture (7:3) and filtered through 0.22 µm polytetrafluoroethylene (PTFE) membrane (Macherey‐Nagel GmbH & Co. KG—Düren, Germany). The HPLC system was equipped with a DGU‐20A5R degasser, LC‐20AT pump, LPGE valve kit, SIL‐20AHT injector, CTO‐20A oven, SPD‐M20A DAD, and CBM‐20A controller (Shimadzu—Kyoto, Japan). Data processing was performed with LabSolutions software. Chromatographic separation was carried out on a Phenomenex Luna C18 column (250 × 4.6 mm; 5 µm) at 40°C. The mobile phase consisted of ultrapure water (phase A) acidified with 1% trifluoroacetic acid (TFA) (Dinâmica—São Paulo, Brazil) and methanol (phase B) at a flow rate of 1 mL/min. A gradient elution program was applied: 0–55 min, 5%–100% B; 55–60 min, 100% B. Analyses were performed in a wavelength scan in a range of 190 to 700 nm and UV detection at 254 nm.

### 4.4 Annotation of the Chemical Constituents by DI‐MS

Both extracts were individually dissolved in methanol (HPLC grade) at a concentration of 0.2 mg mL^−1^ and filtered through 0.22 µm PTFE membranes. A volume of 2.0 mL was measured and introduced using a compatible syringe. The samples were analyzed by DI‐ESI‐IT‐MS^n^ using an AmaZon X mass spectrometer (Bruker Daltonics, Bremen, Germany). For the analysis, the ionization source was set in negative ion mode with an *m/z* range of 100–1500. The syringe flow rate was 5.0 µL/min, the capillary high voltage was 4.5 kV, the nitrogen (N_2_) drying gas flow rate was 4.0 L/min, the nebulizer pressure was 14 psi, and the dry gas source temperature was 230°C. For MS^n^ analysis, signal amplitude, data acquisition time, and radiofrequency parameters were adjusted to optimize spectral information for each precursor ion. Data acquisition and analysis were performed using Bruker Compass Data Analysis software (version 4.3), provided by the manufacturer [10].

### 4.5 Antioxidant Activity

The antioxidant activity of the extracts was evaluated using the DPPH method [12]. A stock solution of DPPH (Sigma‐Aldrich—Poole, UK) at a concentration of 0.04 mg/mL was prepared. Butylhydroxytoluene (BHT) (Sigma‐Aldrich—Poole, UK), a synthetic antioxidant compound, was used as a positive control. Sample solutions were prepared by dissolving 12.0 mg of each extract to obtain stock solutions at 250 µg/mL, which were further diluted to concentrations of 200, 150, 100, 50, and 25 µg/mL. The reaction mixtures consisted of 0.3 mL of the sample solution and 2.7 mL of the DPPH stock solution (40 µg/mL). Absorbance was measured at 516 nm in triplicate after 30 min. Methanol (2.7 mL) combined with 0.3 mL of the extract solution served as the blank. The DPPH concentration was determined using the analytical curve equation A = 33.227c + 1.0607 (*r* = 0.9997), where “c” is the DPPH concentration in the reaction medium, and “A” is the absorbance at a wavelength of 516 nm (λ_max_) measured in a Lambda 25 UV/Vis spectrometer (PerkinElmer—São Paulo, Brazil). From this equation and the absorbance values over 30 min for each tested concentration. The percentage of antioxidant activity (%AA) of the extracts was determined using the absorption of the initial DPPH solution (AbsDPPH), of the reaction mixture with DPPH and sample (Abssample), and blank solution (Absblank), as demonstrated in Equation (1):

(1) $$\begin{array}{ccc} \%\mathrm{AA}\; & = & \left[\mathrm{AbsDPPH}\;-\;\left(\mathrm{Abssample}\;-\;\mathrm{Absblank}\right)\right]/\mathrm{AbsDPPH}\;\\ & & \times \;100 \end{array}$$

To calculate the percentage of remaining DPPH (%DPPH_rem_), the initial concentration of the DPPH solution (DPPH_t = 0_) and the concentration of the reaction mixture with DPPH and sample after 30 min (DPPH_t = 30_), as shown in Equation (2):

(2) $$\%\mathrm{DPP}H_{\mathrm{rem}}=\mathrm{DPP}H_{t=30}/\mathrm{DPP}H_{t=0}\times 100$$

The efficient concentration (EC_50_), defined as the concentration of extract required to reduce the DPPH radical concentration by 50%, was determined in µg/mL from the first‐order exponential curve of %DPPHrem versus sample concentrations.

### 4.6 Cell Culture

The human breast cancer cell line MDA‐MB‐231 (Homo sapiens, female, mammary gland, ATCC HTB‐26) was obtained from the American Type Culture Collection (ATCC, Manassas, VA, USA). This triple‐negative breast cancer cell line was maintained in RPMI‐1640 medium (Gibco, Thermo Fisher Scientific, USA) supplemented with 10% fetal bovine serum (Cultilab, Brazil), 100 U/mL penicillin, 100 µg/mL streptomycin, and 0.25% trypsin–EDTA (all from Sigma‐Aldrich, USA), under standard conditions (37 °C in a humidified atmosphere with 5% CO₂). Cells were subcultured at 70%–80% confluency and seeded at appropriate densities according to each assay. The cell line was tested and confirmed to be free of mycoplasma contamination prior to experimental use.

### 4.7 Cytotoxicity

The cytotoxicity of the extracts was evaluated using the MTT assay (3‐(4,5‐dimethylthiazol‐2‐yl)‐2,5‐diphenyltetrazolium bromide) [18]. For the assay, cells were seeded into 96‐well plates (3 × 10^3^ cells/100 µL) and allowed to adhere for 24 h under the described culture conditions. Subsequently, cells were exposed to different extract concentrations (1.5–800 µg/mL) for 72 h. After exposure, 100 µL/well of MTT solution (1 mg/mL) was added, and the plates were incubated for 4 h, protected from light. The medium was then replaced with 100 µL/well of DMSO to solubilize the formazan crystals. Absorbance was measured at 570 nm using a microplate reader. The cytotoxic concentration required to inhibit 50% of the cell population (CC_50_) was calculated.

### 4.8 Cell Viability (Live and Dead Assay)

Cell viability was assessed using fluorescence‐based labeling. MDA‐MB‐231 cells were cultured as described above and plated in 96‐well plates at a density of 3 × 10^3^ cells/100 µL. After 24 h, the cells were treated with the extracts at concentrations of 1, 10, and 100 µg/mL for 72 h. Following treatment, the culture medium was removed, and the cells were washed with 100 µL of sterile PBS. The cells were labeled with 1.25 µL of Calcein AM (0.01 M) to mark viable cells and 1.25 µL of Hoechst 33342 (2 mg/mL) to stain nuclei. The plates were incubated for 20 min at 37°C. After washing with PBS, cells were incubated for 5 min with 50 µL of PI (1 mg/mL) to stain dead cells. Following a final PBS wash, fluorescence was captured using a fluorescence microscope (Evos) at excitation/emission wavelengths of 488 nm (Calcein‐AM), 570 nm (PI), and 345 nm (Hoechst 33342). The results were expressed as the percentage of fluorescence of viable (Calcein‐AM) or dead (PI) cells normalized to the total number of cells in each treatment condition (Hoechst 33342) [19].

### 4.9 Genotoxicity

DNA damage in MDA cell lines was assessed using the comet assay [20]. Cells were seeded in 24‐well plates and, after 24 h, exposed to treatments in triplicate. Following 24 h of treatment, cells were detached using a 0.25% trypsin solution, and 10 µL of the cell suspension from each treatment was mixed with 90 µL of low‐melting point agarose (0.75%). This mixture was applied onto slides pre‐coated with normal‐melting agarose and left for 72 h at 4°C in a lysis solution (2.5 M NaCl, 100 mM EDTA, 10 mM Tris, pH 10, with 1% Triton X‐100 and 10% DMSO). After lysis, the slides were immersed in an alkaline buffer (300 mM NaOH and 1 mM EDTA, pH > 13) for 20 min. Electrophoresis was performed under 300 mA and 25 V (0.90 V/cm) for 15 min. Subsequently, the slides were neutralized with Tris buffer (0.4 M, pH 7.5) and stained with a silver solution. The cells were then evaluated for their photomicrographic profile using an optical microscope at 400x magnification. DNA damage was categorized into classes ranging from 0 to 4. The results were expressed as the damage index (DI) and damage frequency (DF) based on the evaluation of 100 cells. DI was calculated using the formula: DI = Σ (number of cells in a given damage class × damage class) with values ranging from 0 to 400. DF was determined using the formula: DF = 100 − (percentage of cells with damage class 0).

### 4.10 Antitumor Activity in TNBC Model

A suspension of 4T1 TNBC cells (5 × 10^6^ cells/100 µL PBS) was subcutaneously inoculated into the left mammary fat pad of Balb/c mice (7 animals per group). Twenty‐four h post‐inoculation, the treatment groups received HAFR at doses of 0.03, 0.3, and 3 g/kg orally for 21 days. Doxorubicin was administered as a positive control at 37.5 mg/kg intraperitoneally, in four doses at 5‐day intervals. At the end of the treatment period, the animals were euthanized. The doses were selected based on previous toxicity studies. At the end of the treatment, the body weight of the animals, liver weight, and tumor weight were recorded for analysis. All animal handling and experimental procedures were conducted with prior approval by the Ethics Committee in Animal Experimentation at the Federal University of Piaui (# 772/2023, August 09, 2024) and in accordance with the American Veterinary Medical Association (AVMA) Guidelines for the Euthanasia of Animals (2020) and ARRIVE guidelines [21].

### 4.11 Determination of MDA

Mouse livers were homogenized at a ratio of 100 mg tissue per 1 mL of KCl solution (0.15 M) and centrifuged at 1500 g for 10 min at 4°C. The supernatant was collected, and 100 µL was mixed with 100 µL of 8.1% sodium dodecyl sulfate, 800 µL of 3.5 M sodium acetate, and 800 µL of 0.8% thiobarbituric acid. The mixture was vortexed and incubated at 95°C for 1 h, followed by cooling in an ice bath for 5 min. Samples were then centrifuged at 1500 g for 10 min at 4°C, and the absorbance of the supernatant was measured at 532 nm. A standard curve was prepared using 4 mM TEP (1,1,3,3‐tetramethoxypropane) diluted in KCl (0.15 M) at concentrations of 0, 12.5, 25, 50, 100, 200, and 400 µM. Each standard was treated identically to the samples, and KCl served as the blank. Results were expressed in µmol of MDA, calculated based on the standard curve [22].

## 5 Statistical Analysis

Results were expressed as mean ± standard error of the mean. Statistical analyses were conducted using one‐way ANOVA followed by Bonferroni's post‐hoc test. For the comet assay, values were expressed as mean ± standard deviation and analyzed using two‐way ANOVA with Tukey's post‐hoc test. A *p* < 0.05 was considered statistically significant.

## Author Contributions

**Diego Pereira de Menezes**: conceptualization, data curation, formal analysis, acquisition of financing, investigation, resources, and writing – original draft. **Evaldo dos Santos Monção**: formal analysis and methodology. **Atanara Alves de Sousa**: methodology. **João Pedro Alves Damasceno do Lagod**: methodology. **Karla Darlianny Mourão Cavalcante**: methodology. **Fernanda Alves de Sousa Cavalcante**: methodology. **Irismara Sousa Silva**: conceptualization, formal analysis, and supervision. **Marina Pinto Sanches**: data curation. **Semiramis Jamil Hadad do Monte**: formal analysis and methodology. **Ester Miranda Pereira**: formal analysis and methodology. **Paulo Michel Pinheiro Ferreira**: conceptualization, supervision, and validation**. João Marcelo de Castro e Sousa**: conceptualization, formal analysis, supervision, and validation. **Gerardo Magela Vieira Júnior**: conceptualization, formal analysis, supervision, and validation. **Dalton Dittz**: conceptualization, formal analysis, investigation, methodology, project administration, resources, supervision, validation, and writing – review and editing.

## Conflicts of Interest

The authors declare no conflicts of interest.

## Supporting information




**Supporting File 1**: cbdv70218‐sup‐0001‐SuppMat.pdf

## Data Availability

The data that support the findings of this study are available from the corresponding author upon reasonable request.
