# Supplementary material for: Antitumor and Cytogenotoxic Activities of Libidibia ferrea Hydroalcoholic Extracts in Murine Breast Carcinoma
Source: Chem Biodivers. 2025 Jul 11;22(11):e01032. doi: 10.1002/cbdv.202501032 (PMC12629168; doi:10.1002/cbdv.202501032)
Supplement: Supplementary file 1 — Supporting File 1: cbdv70218‐sup‐0001‐SuppMat.pdf [file CBDV-22-e01032-s001.pdf]

Supporting Information

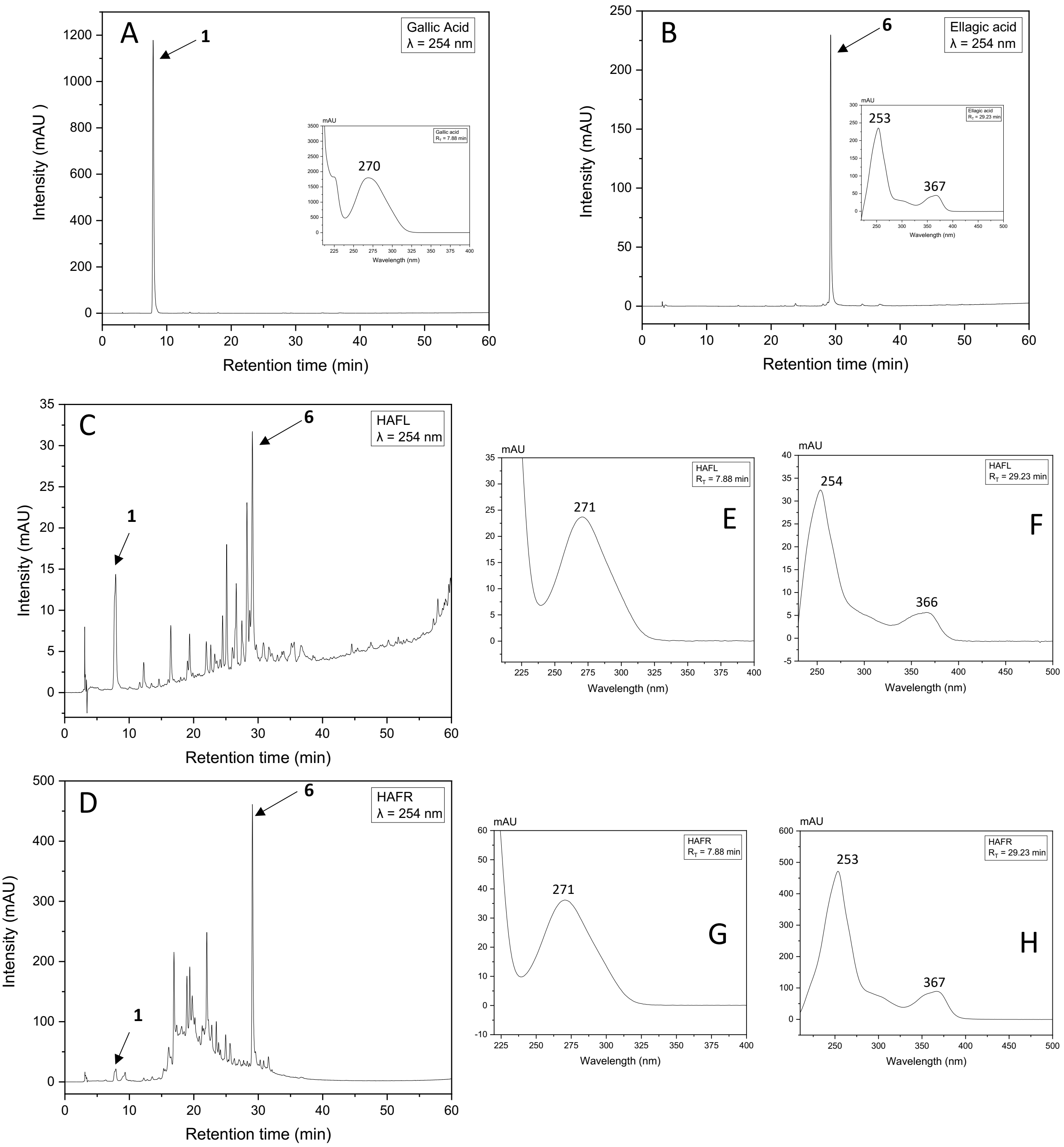

Figure S1. Chromatograms and UV spectrum by HPLC-DAD of gallic acid (**1**) and ellagic acid (**6**) standards and HAFL and HAFR extracts: gallic acid (**A**) and ellagic acid (**B**) chromatograms and UV Spectrum. HAFL (**C**) and HAFR (**D**) extracts chromatograms and (**E**), (**F**), (**G**) and (**H**) their respective UV spectrum in comparison at same retention time (Rt) of standards.

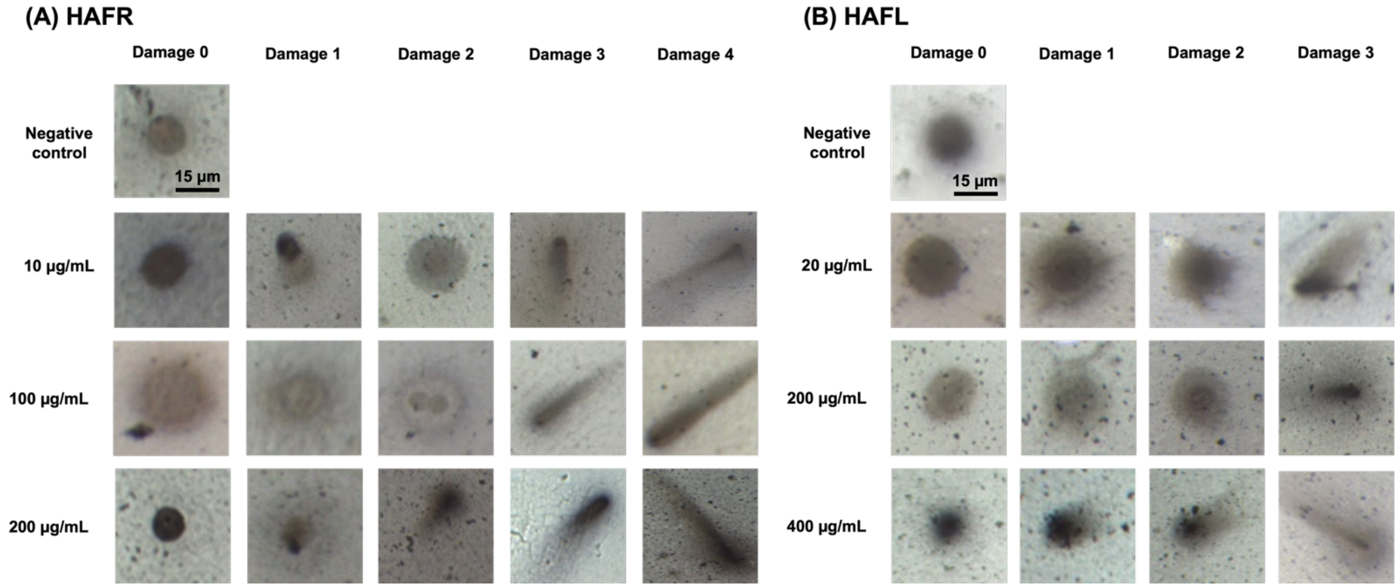

Figure S2. Representative images of nuclear DNA damage in MDA-MB-231 cells induced by treatment with HAFR (**A**) and HAFL (**B**) extracts. The images illustrate typical patterns of genetic damage used for scoring damage index (D.I.) and damage frequency (D.F.), including tail formation and fragmentation observed in the comet assay.
